# Prostate cancer ETS rearrangements switch a cell migration gene expression program from RAS/ERK to PI3K/AKT regulation

**DOI:** 10.1186/1476-4598-13-61

**Published:** 2014-03-19

**Authors:** Nagarathinam Selvaraj, Justin A Budka, Mary W Ferris, Travis J Jerde, Peter C Hollenhorst

**Affiliations:** 1 Medical Sciences, Indiana University School of Medicine, 1001 E 3rd St, Bloomington, IN 47405, USA; 2 Pharmacology and Toxicology, Indiana University School of Medicine, Indianapolis, IN 46202, USA

**Keywords:** Prostate cancer, ETS, RAS/ERK, PI3K/AKT, Cell migration

## Abstract

**Background:**

The RAS/ERK and PI3K/AKT pathways induce oncogenic gene expression programs and are commonly activated together in cancer cells. Often, RAS/ERK signaling is activated by mutation of the *RAS* or *RAF* oncogenes, and PI3K/AKT is activated by loss of the tumor suppressor *PTEN*. In prostate cancer, *PTEN* deletions are common, but, unlike other carcinomas, *RAS* and *RAF* mutations are rare. We have previously shown that over-expression of “oncogenic” ETS transcription factors, which occurs in about one-half of prostate tumors due to chromosome rearrangement, can bypass the need for RAS/ERK signaling in the activation of a cell migration gene expression program. In this study we test the role of RAS/ERK and PI3K/AKT signaling in the function of oncogenic ETS proteins.

**Results:**

We find that oncogenic ETS expression negatively correlates with *RAS* and *RAF* mutations in prostate tumors. Furthermore, the oncogenic ETS transcription factors only increased cell migration in the absence of RAS/ERK activation. In contrast to RAS/ERK, it has been reported that oncogenic ETS expression positively correlates with PI3K/AKT activation. We identified a mechanistic explanation for this finding by showing that oncogenic ETS proteins required AKT signaling to activate a cell migration gene expression program through ETS/AP-1 binding sequences. Levels of pAKT correlated with the ability of oncogenic ETS proteins to increase cell migration, but this process did not require mTORC1.

**Conclusions:**

Our findings indicate that oncogenic ETS rearrangements cause a cell migration gene expression program to switch from RAS/ERK control to PI3K/AKT control and provide a possible explanation for the high frequency of *PTEN*, but not *RAS/RAF* mutations in prostate cancer.

## Background

The RAS/RAF/MEK/ERK (RAS/ERK) and PI3K/AKT signaling pathways regulate gene expression programs that promote cell growth, proliferation, motility, and survival [1,2]. Mutations that cause constitutive RAS/ERK or PI3K/AKT signaling are among the most common alterations in human cancer and both pathways are often activated in the same tumor [3,4]. PI3K/AKT activation is common in prostate cancer, often due to loss of a suppressor of the pathway, PTEN [5]. However, unlike other carcinomas, prostate cancers rarely have activating mutations in RAS or RAF [6], and thus, the mechanisms that allow transcriptional activation of RAS/ERK target genes in this malignancy are not fully understood.

RAS/ERK signaling can be initiated by tyrosine kinase receptors that activate RAS, followed by the RAF/MEK/ERK kinase cascade, resulting in phosphorylated ERK (pERK). pERK, in turn, phosphorylates transcription factors, including some members of the ETS family, leading to increased transcriptional activation of target genes [7]. PI3K phosphorylates phosphoinositides leading to activation of downstream proteins such as the kinase AKT [8]. PTEN, a phosphatase, can reverse this process and acts as a tumor suppressor. Activated AKT has multiple functions, one being the activation of the mTOR containing signaling complex mTORC1, which alters translational control of gene expression. AKT also activates the mTORC2 complex, which provides positive feedback by phosphorylating and activating AKT. The RAS/ERK and PI3K/AKT pathways are highly interconnected. For example, RAS can activate PI3K, and AKT can phosphorylate and inhibit RAF [9,10].

A rearrangement of chromosome 21 that results in fusion of the *TMPRSS2* and *ERG* genes occurs in approximately 50% of prostate tumors [11]. *TMPRSS2:ERG* joins the 5′ regulatory regions and 5′ UTR of *TMPRSS2*, which is highly expressed in prostate, to the open reading frame of *ERG*, resulting in expression of either a full-length, or N-terminally truncated version of *ERG*, an ETS family transcription factor that is not normally expressed in prostate cells. Similar fusions that over-express the ETS genes *ETV1*, *ETV4*, and *ETV5* occur in another 10% of prostate tumors [11-13]. Expression of these oncogenic ETS family members in prostate cells drives cellular invasion and migration [14,15] and promotes the transition from neoplasia to carcinoma [16]. We previously reported that over-expression of *ERG* or *ETV1* can activate a gene expression program that drives cell migration [15]. Genes in this program are regulated by a RAS-responsive enhancer sequence consisting of neighboring ETS and AP-1 transcription factor binding sites. In normal prostate cells, these genes can be activated by RAS/ERK signaling, likely via ERK phosphorylation of an ETS protein bound to the ETS/AP-1 sequence. There are 12–15 ETS transcription factors expressed in normal prostate that are candidates for this role [17]. Our previous data support a model that when ERG, ETV1, ETV4, or ETV5 are over-expressed in prostate cells, they can replace the ETS family member(s) normally bound to ETS/AP-1 sites and activate the RAS-inducible cell migration gene expression program in the absence of RAS/ERK signaling [15]. Thus over expression of one of these four “oncogenic” ETS genes can mimic RAS/ERK pathway activation.

The two most common genomic aberrations in prostate cancer are *PTEN* deletion and the *TMPRSS2/ERG* rearrangement [11,18,19]. Whereas a RAS mutation in other carcinomas might activate both ERK and PI3K signaling, we propose that prostate tumors have an alternative way to activate these pathways: *PTEN* deletion (PI3K/AKT activation) coupled with oncogenic ETS-overexpression (activation of RAS/ERK target genes). Supporting this hypothesis, *PTEN* deletion is more common in prostate tumors with *TMPRSS2-ERG* rearrangements, than in those without [16,20], and in mouse models, *ERG* over-expression results in adenocarcinoma only when accompanied by a second mutation that activates the PI3K/AKT pathway [16,20,21].

Here we test the relationship between oncogenic ETS expression and both the RAS/ERK and PI3K/AKT pathways. We provide the first comprehensive analysis of oncogenic ETS protein expression in prostate cancer cell lines. We then show that the status of both the RAS/ERK and PI3K/AKT pathways can change the ability of over-expressed ETS proteins to promote prostate cell migration. Significantly, we find that oncogenic ETS expression makes cell migration less dependent on RAS/ERK signaling, but increases the importance of PI3K/AKT signaling. We provide evidence that this switch in the signaling pathway requirement is due to AKT-dependent, but mTORC1-independent, regulation of oncogenic ETS function through ETS/AP-1 binding sequences. Therefore, switching the ETS protein at ETS/AP-1 sequences changes the ability of signaling pathways to regulate a critical oncogenic gene expression program.

## Results

### Oncogenic ETS gene rearrangement occurs in tumors lacking RAS/ERK mutations

If oncogenic ETS gene rearrangements replace RAS/ERK activation, we predict that RAS/ERK mutations will occur only in ETS rearrangement negative tumors. To test this hypothesis, we examined the results of three recently published studies [6,22,23] that both sequence exons and identify chromosome rearrangements in prostate tumors (Table 1). Together these studies examine 266 prostate tumors. One-half (133) have ERG or ETV1 chromosome rearrangements. We searched for either gene fusions, or point mutations in canonical RAS/ERK pathway genes (RAS, RAF, MEK, and ERK encoding genes). Eight tumors had such aberrations, and all eight were negative for oncogenic ETS rearrangements. This indicates that, while genomic alterations in RAS/ERK pathway components are rare in prostate cancer, there is a statistically significant (P = 0.007; Fisher’s exact test) mutual exclusivity of these alterations and ETS rearrangements. It has been previously reported that PI3K/AKT activation via PTEN deletion positively correlates with ETS gene rearrangements [16,20]. A search for PTEN loss in these 266 tumors (Table 1) confirms these findings and indicates that PTEN loss is more than twice as likely in tumors with ETS gene rearrangements than in those without (P = 0.0008; Fisher’s exact test). In conclusion, *ERG* and *ETV1* gene rearrangements positively correlate with PTEN loss and negatively correlate with RAS/ERK mutations in tumors.

**Table 1:** **Correlation of RAS/ERK pathway mutations, *PTEN *** **loss, and oncogenic ETS expression in prostate tumors**

	**ETS -**	**ETS +**	**Total**
PTEN loss	19	43	62
RAS/ERK mutation	8	0	8
APC mutation	6	5	11
Total	133	133	266

A summary of the 266 tumors analyzed by Taylor *et al.*[22], Grasso *et al*. [6], and Baca *et al*. [23]. ETS status indicates presence (+) or absence (-) of an *ERG* rearrangement in Taylor *et al.* and either an *ERG* or *ETV1* rearrangement in Grasso *et al.* and Baca *et al*. RAS/ERK mutation includes point mutations from all three studies or verified gene rearrangements resulting in transcript fusions in Grasso *et al.* or Baca *et al*. for HRAS, KRAS, NRAS, RAF1, ARAF, BRAF, MAP2K1, MAP2K2, MAPK1, or MAPK3. APC mutations are included as a control to show that low frequency mutations are not always enriched in one category.

### Prostate cancer cell lines as models of oncogenic ETS function

**Figure 1:**
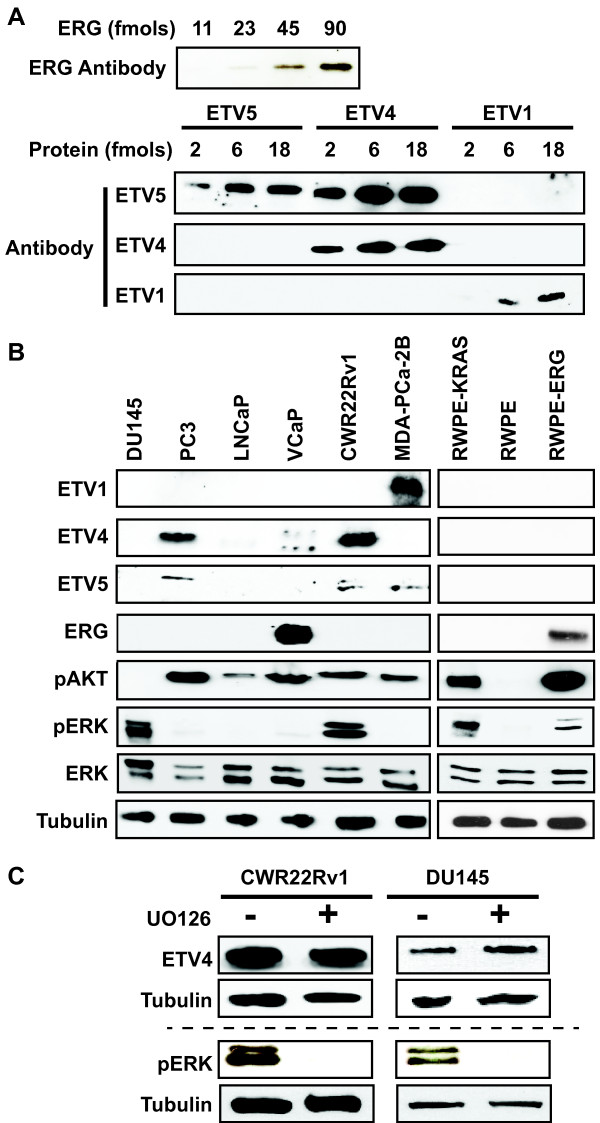
**Prostate cell lines vary in oncogenic ETS expression and RAS/ERK pathway activation. (A)** The sensitivity and specificity of antibodies detecting oncogenic ETS proteins were tested by immunoblot of the indicated amount of purified full-length proteins. **(B)** Immunoblots show levels of four oncogenic ETS proteins, pAKT (PI3K/AKT activation), pERK (RAS/ERK activation), total ERK, and tubulin control in six prostate cancer cell lines (left) and three cell lines derived from normal prostate (right). **(C)** Immunoblots show levels of ETV4 and pERK in the indicated cell lines with or without U0126 (10 μM, 10 hr). The same cell extracts are loaded on one gel above the dashed line, and a second gel below. ETV4 is only visible in DU145 cells after a very long exposure, hence it is not observed in **(A)**.

To test the effect of RAS/ERK signaling and PI3K/AKT signaling on oncogenic ETS function in prostate cell lines, we must first determine which cell lines have these characteristics. Although some prostate cancer cell lines, such as VCaP (*ERG*) and LNCaP (*ETV1*) are reported to have oncogenic ETS gene rearrangements [11,14], the full extent of oncogenic ETS protein expression, including fusion-independent expression, in commonly used prostate cancer cell lines has not been determined. To identify the expression level of the four oncogenic ETS proteins, we first tested available antibodies using purified recombinant proteins (Figure 1A). We identified antibodies to ERG, ETV1, ETV4, and ETV5 that could detect each protein at femtomolar levels. Because ETV1, ETV4, and ETV5 are homologous proteins, the sensitivity and specificity of these antibodies were compared. ETV1 and ETV4 antibodies were specific, but the ETV5 antibody recognized ETV4 and ETV5 equally. We then examined oncogenic ETS protein levels, along with phosphorylated ERK (pERK: RAS/ERK pathway) and phosphorylated AKT (pAKT: PI3K/AKT pathway) levels in six prostate cancer cell lines (Figure 1B and Additional file 1: Figure S1A). DU145 cells, which have a KRAS gene rearrangement [24], did not have high levels of any oncogenic ETS protein, or pAKT, but did have pERK, consistent with the small fraction of prostate cancers with RAS/ERK pathway mutations (Table 1). Of the remaining five prostate cancer cell lines, four had high expression of a single oncogenic protein. These included ERG in VCaP, consistent with a *TMRPSS2/ERG* rearrangement [11], ETV1 in MDA-PCa-2B, consistent with an *ETV1* gene rearrangement [14], and ETV4 in PC3, consistent with high *ETV4* mRNA [25]. ETV4 protein was also present at high levels in CWR22Rv1. Of the four lines with high oncogenic ETS protein expression, all had high levels of pAKT, but only one (CWR22Rv1) had high levels of pERK, consistent with the analysis of prostate tumors in Table 1. Surprisingly, despite an ETV1 gene rearrangement [14], and high ETV1 mRNA levels [25], ETV1 protein was not observed in LNCaP cells. However, this is consistent with results from Vitari *et al.* who showed low ETV1 protein levels in LNCaP cells due to proteasomal targeting by the COP1 E3 ubiquitin ligase [26].

Long exposures could identify pERK, pAKT, and some ETS proteins at low levels in immunoblots from most cell lines. To more quantitatively establish the “high-level” threshold shown in Figure 1B, ETS proteins in cell extracts were compared with purified standards (Additional file 1: Figure S1B). All “high-level” expression for ETS proteins exceeded 50,000 proteins per cell, and was highest at 330,000 proteins per cell for ERG in VCaP. Low-level ETS expression was 10,000 proteins per cell (ETV4 in DU145) or less (Additional file 1: Figure S1B and data not shown).

It is possible that oncogenic ETS expression and signaling pathway activation could influence each other. To test this, RWPE-1 (RWPE) cells derived from normal prostate [27] or variations of this line that express either Ki-RAS (RWPE-KRAS, also known as RWPE-2) or ERG (RWPE-ERG) were compared. ERG levels in RWPE-ERG cells were similar to VCaP cells (Additional file 1: Figure S1C). None of the oncogenic ETS were expressed at high levels in RWPE or RWPE-KRAS cells, and only ERG was expressed in RWPE-ERG cells (Figure 1B). As expected, KRAS increased both pERK and pAKT levels (Figure 1B). Interestingly, over-expression of ERG also resulted in activation of AKT and a small increase in pERK (Figure 1B). In other cell types, the RAS/ERK pathway activates ETV1, ETV4, and ETV5 expression [28]. Therefore, high ETV4 expression in CWR22Rv1 cells could be the result of ERK activation. To test this, CWR22Rv1 and DU145 cells were treated with the MEK inhibitor U0126 for 24 hours. In both cell lines, U0126 decreased pERK levels, but did not alter levels of ETV4 (Figure 1C). Therefore, RAS/ERK activation does not drive oncogenic ETS expression in prostate cancer cell lines, however in at least one context (ERG in RWPE) an oncogenic ETS could induce the phosphorylation of both AKT and, to a lesser degree, ERK.

### Oncogenic ETS proteins and KRAS drive prostate cell migration, but not synergistically

**Figure 2:**
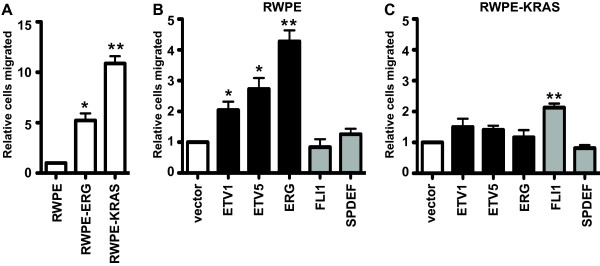
**ETS expression and RAS activation induce migration of prostate cells via the same pathway. (A)** A transwell assay measured relative number of migrating RWPE cells expressing ERG or activated KRAS relative to normal RWPE cells (first lane). **(B, C)** Transwell assays measured migration of **(B)** RWPE cells, or **(C)** RWPE-KRAS cells expressing oncogenic (Black bars) or non-oncogenic (Grey bars) ETS proteins. Number of cells migrated is reported relative to the same cell line transduced with an empty vector (white bar). Mean and SEM of three biological replicates (each mean of two technical replicates) are shown for **(A)** and five biological replicates for **(B)** and **(C)**. P-values compare indicated value to the hypothetical mean (1) and are calculated by t test: * < 0.05, ** < 0.005, unmarked > 0.05.

We next tested the role of signaling pathways in the ability of oncogenic ETS proteins to drive cell migration. Because cancer derived cell lines have many mutations and copy number alterations that affect cellular phenotypes, we used the RWPE-ERG and RWPE-KRAS cell lines to compare the ability of oncogenic ETS and RAS signaling to promote cell migration in the same cellular background. RWPE-ERG and RWPE-KRAS cells migrated 5- and 10-fold more than RWPE cells (Figure 2A and Additional file 2: Figure S2), indicating that both ERG and KRAS induce cell migration. Similar to our previous findings [15], overexpression of oncogenic ETS proteins ETV1, ETV5, and ERG, but not other ETS proteins (FLI1 and SPDEF), promoted RWPE cell migration (Figure 2B and Additional file 2: Figure S2). In contrast, when the same ETS proteins were over-expressed in RWPE-KRAS cells, none of the oncogenic ETS proteins induced additional cell migration (Figure 2C and Additional file 2: Figure S2), suggesting that these ETS proteins and KRAS were functioning to activate the same pathway. These findings are consistent with our model that oncogenic ETS proteins can mimic RAS activation in cell lines lacking RAS activity, and are distinct from ETS proteins expressed in normal prostate.

### A role for the PI3K/AKT pathway in oncogenic ETS function

To identify signaling pathways required for the oncogenic function of ETS factors, a microarray analysis of *ETV4* knockdown in PC3 prostate cancer cells [25] was compared to the Connectivity Map database [29] that contains microarray data of PC3 cells treated with 1309 small molecules, including many signaling pathway inhibitors. Similarities between the gene expression profile of a signaling pathway inhibitor and *ETV4* knockdown would predict a role for that pathway in oncogenic ETS function. The top two, and three of the top five small molecules that induced gene expression changes most similar to *ETV4* knockdown were inhibitors of either PI3K or mTOR, a downstream effector of PI3K (Table 2). These data suggest that in PC3 cells, PI3K and ETV4 activate a similar gene expression program.

**Table 2:** **Drugs that alter PC3 gene expression most similarly to *ETV4 *** **depletion**

**Rank**	**Drug**	**Target**	**P value**
1	Sirolimus (Rapamycin)	mTOR	<0.00001
2	LY-294002	PI3K	<0.00001
3	Trichostatin A	HDAC	0.00002
4	Alexidine	Tyrosine Phosphatase	0.00004
5	Wortmannin	PI3K	0.00006

Gene expression changes from small molecule treatments of PC3 cells in the Connectivity Map database [29] were compared to gene expression changes previously reported for ETV4 depletion in PC3 cells [25]. Small molecules that elucidated changes most similar to ETV4 depletion are rank ordered by P value.

**Figure 3:**
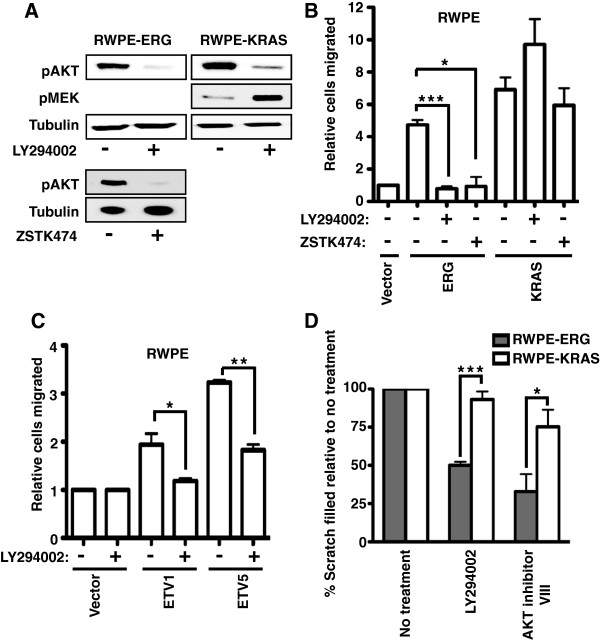
**An active PI3K/AKT pathway is required for oncogenic ETS, but not KRAS, to induce prostate cell migration. (A)** An immunoblot shows the levels of pAKT, pMEK (activator of ERK), or tubulin (control) after LY294002 (20 μM; 24 h) or ZSTK474 (2 μM; 24 h) treatment in RWPE-ERG or RWPE-KRAS cells. **(B)** A transwell assay measured cell migration of RWPE prostate cells with or without ERG and KRAS overexpression and in the presence or absence of the PI3K inhibitors LY294002 (20 μM) or ZSTK474 (2 μM). The number of migrated cells is shown as the mean and SEM of six biological replicates (except for ZSTK474 treated cells which have three replicates) relative to RWPE-empty vector. **(C)** A transwell assay, as in **(A)**, tested the role of PI3K inhibition on ETV1 and ETV5 expressing RWPE cells and shows the mean and SEM of three biological replicates. **(D)** Results of the scratch assay performed in the presence or absence of LY294002 (20 μM) and AKT inhibitor VIII (10 μM) in RWPE-ERG (Grey bar) and RWPE-KRAS (white bar) cells. The percentage of scratch filled is shown as the mean and SEM of three biological replicates (each mean of three technical replicates) relative to no treatment. P-values are calculated by t test: * < 0.05, ** < 0.005, *** < 0.0005, unmarked > 0.05.

To test if the PI3K pathway is required for an oncogenic ETS protein to promote the cell migration phenotype, RWPE-ERG and RWPE-KRAS cells were treated with the PI3K inhibitor, LY294002. LY294002 reduced AKT phosphorylation in both lines, consistent with PI3K inhibition (Figure 3A). Strikingly, PI3K inhibition completely abrogated cell migration induced by ERG, but not cell migration induced by KRAS (Figure 3B and Additional file 2: Figure S2). In fact RWPE-KRAS cells actually migrated more when PI3K was inhibited. This increased migration may be due to relief of RAF inhibition by AKT [9], as RWPE-KRAS cells had higher pMEK levels after treatment by LY294002 (Figure 3A). To confirm the role of PI3K, a second PI3K inhibitor, ZSTK474, was also tested (Figures 3A and 3B). Like LY294002, ZSTK474 significantly reduced migration of RWPE-ERG cells, but not RWPE-KRAS cells. Cell migration induced by other oncogenic ETS factors, ETV1, and ETV5, was also abrogated by PI3K inhibition (Figure 3C and Additional file 2: Figure S2). A second cell migration assay, the scratch assay, confirmed that PI3K inhibition reduced migration caused by ERG expression, but not migration caused by KRAS (Figure 3D and Additional file 3: Figure S3). An AKT inhibitor had a similar effect (Figure 3D and Additional file 3: Figure S3), indicating that PI3K is functioning via AKT activation. These results indicate that overexpression of an oncogenic ETS gene can switch the control of prostate cell migration from the RAS/ERK pathway to the PI3K/AKT pathway.

**Figure 4:**
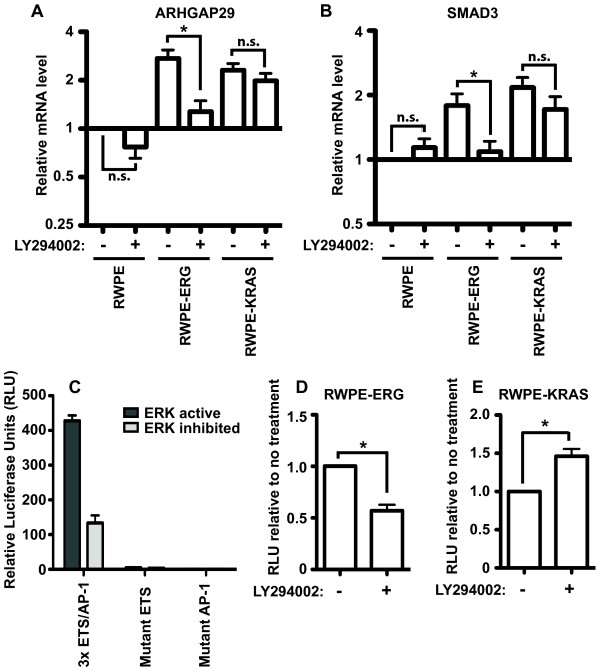
**The PI3K pathway can alter the expression of cell migration genes via ETS/AP-1 sites in oncogenic ETS overexpressing cells.** mRNA expression of **(A)***ARHGAP29* or **(B)***SMAD3*, in the presence and absence of PI3K inhibitor (LY294002, 20 μM), in RWPE-ERG and RWPE-KRAS cells was measured by qRT-PCR and compared to RWPE cells. Mean and SEM of seven biological replicates are shown. **(C)** Firefly luciferase activity from a vector with the indicated sequences (3 copies of neighboring ETS and AP-1 binding sequences or versions of the same with point mutations) is shown relative to Renilla luciferase from a control vector transfected in RWPE cells. The ERK pathway is inhibited by UO126 where indicated. Mean and SEM of six biological replicates (each mean of two technical replicates) are shown. Luciferase reporter activity measured as in **(C)** is shown in **(D)** RWPE-ERG, or **(E)** RWPE-KRAS cells as activity in LY294002 treated cells (20 μM) relative to untreated. P-values are calculated by t test: n.s > 0.10, * < 0.05.

We next tested if the PI3K pathway was regulating the ability of ERG to activate the transcription of RAS- and ERG-responsive target genes near enhancers that are co-occupied by ETS and AP-1 proteins. The expression levels of two such genes, *ARHGAP29*, and *SMAD3,* were measured by quantitative reverse transcription PCR (qRT-PCR) (Figure 4A and B). Both *ARHGAP29* and *SMAD3* have roles in cell migration and/or cell morphology [30,31], are direct targets of oncogenic ETS proteins and AP-1 by ChIP-seq [15], and are activated by KRAS and oncogenic ETS expression (Figure 4A and B). Similar to the cell migration phenotype, the activation of both genes was significantly attenuated by PI3K inhibition in RWPE-ERG cells, but not in RWPE-KRAS cells (Figure 4A and B). Therefore cell migration changes are consistent with changes in the expression of these two oncogenic ETS target genes.

These results indicate that the PI3K/AKT pathway functions through ERG to regulate expression of cell migration genes. We next used a reporter assay to test if these gene expression changes were mediated by the ETS/AP-1 binding sequences we found in the enhancers of oncogenic ETS target genes. Three copies of an ETS/AP-1 consensus sequence were cloned upstream of a minimal promoter driving firefly luciferase. Luciferase expression from this vector was higher when the ERK pathway was active, indicating that this pathway regulates the reporter construct (Figure 4C). Point mutations in either the ETS or AP-1 binding sequences completely eliminated luciferase expression indicating that both binding sites are required for activity (Figure 4C). The PI3K inhibitor, LY294002, caused a significant decrease in the activity of this reporter in RWPE-ERG cells (Figure 4D), but significantly increased activity in RWPE-KRAS cells (Figure 4E), consistent with the cell migration findings. Therefore, the PI3K pathway can alter the expression of cell migration genes via ETS/AP-1 sites.

### The role of AKT in oncogenic ETS function is not via mTORC1

**Figure 5:**
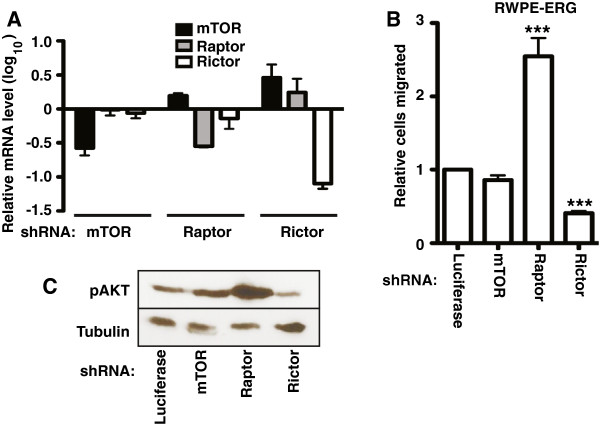
**PI3K/AKT signaling in oncogenic ETS function is not through mTORC1. (A)** shRNA knock down of mTOR, Raptor (mTORC1 complex) and Rictor (mTORC2 complex) in RWPE-ERG cells was confirmed by qRT-PCR analysis. Mean and SEM of two biological replicates (each mean of two technical replicates) are shown. **(B)** A transwell assay measured cell migration of RWPE-ERG cells stably expressing the indicated shRNA relative to a negative control (shRNA targeting luciferase, which is not expressed in this cell line). Results are the mean and SEM of four independent experiments, each the mean of two technical replicates. **(C)** Immunoblot showing the expression level of pAKT and tubulin in RWPE-ERG cells expressing the indicated shRNA. P-values are calculated by t test: *** < 0.0005.

PI3K/AKT signaling has a number of cellular functions including the activation of the mTOR-containing complexes mTORC1 and mTORC2 [8]. mTORC1 includes the Raptor protein and regulates gene expression via translational control. mTORC2 includes the Rictor protein and provides positive feedback by phosphorylating and activating AKT. To test the role of mTOR-containing complexes in oncogenic ETS function, shRNAs were used to knockdown mTOR, Raptor, and Rictor, in RWPE-ERG cells (Figure 5A). Loss of Raptor resulted in an increase in cell migration, indicating that mTORC1 is not required for the ability of PI3K/AKT to promote cell migration (Figure 5B and Additional file 2: Figure S2). Loss of mTOR had little effect on RWPE-ERG migration, while loss of Rictor decreased migration (Figure 5B and Additional file 2: Figure S2). Because the major role of the Rictor-containing mTORC2 complex is thought to be the phosphorylation of AKT, we hypothesized that these results were due to changes in AKT phosphorylation. Consistent with previous findings [32-34], Raptor knockdown increased AKT phosphorylation, and Rictor knockdown decreased AKT phosphorylation (Figure 5C). Therefore, the effect of mTOR containing complexes on RWPE-ERG cell migration can be explained indirectly by changes to pAKT levels, rather than by a direct role.

## Discussion

*PTEN* deletion and the *TMPRSS2:ERG* rearrangement are the two most common genomic aberrations in prostate tumors. These alterations result in activation of the PI3K/AKT pathway and expression of the transcription factor ERG in prostate cells. Expression of ERG alone in prostate epithelia does not induce adenocarcinoma, but ERG is oncogenic when expressed in combination with PI3K/AKT activation [16,20,21], indicating an important synergy between these pathways. Our results identify a mechanistic connection between the expression of oncogenic ETS, such as ERG, and activation of the PI3K/AKT pathway. We show that AKT activation is required for oncogenic ETS proteins to increase transcription of genes critical for cellular migration - a pathway that promotes progression of a neoplasia to an adenocarcinoma. Interestingly, in cells lacking oncogenic ETS expression, these genes are activated by the RAS/ERK pathway through enhancer ETS/AP-1 binding motifs, and are likely activated by mutations in this pathway in other cancers. We show that oncogenic ETS protein expression replaces RAS/ERK regulation of these genes with PI3K/AKT regulation. Our results are consistent with a recent finding that in mice the over-expression of ERG in prostate epithelia only results in significant changes in gene expression when PTEN is deleted [35]. Together these findings provide an explanation for why the PI3K/AKT pathway is activated more often than the RAS/ERK pathway in prostate cancers, but not in other carcinomas that lack ETS gene fusions.

We provide the first comprehensive analysis of oncogenic ETS, pERK and pAKT protein levels in prostate cancer cell lines (Figure 1B). These results indicate that commonly used prostate cancer cell lines recapitulate patterns of oncogenic ETS expression observed in tumors, such as a positive correlation between oncogenic ETS expression and PI3K/AKT pathway activation, and negative correlation between oncogenic ETS expression and RAS/ERK pathway mutations. CWR22Rv1 provided one exception to these correlations, as it expressed ETV4, pERK, and pAKT. This may reflect a unique role for ETV4, since a recent report indicates that expression of ETV4, but not other oncogenic ETS genes correlates with both PI3K and RAS signaling in prostate tumors [36]. Prostate tumors rarely have multiple ETS gene rearrangements [37], leading to the hypothesis that oncogenic ETS genes have overlapping functions and therefore there is no advantage to the tumor to express more than one. Figure 1 indicates that oncogenic ETS proteins, even when expressed in a fusion-independent manner, show the same pattern, supporting this redundancy model. This analysis also revealed that ERG expression strongly increased pAKT levels, which may provide a positive-feedback loop by increasing ERG function (Figure 1B). This contrasts with findings in mice, where ERG did not increase pAKT [16]. It may be that the effect of ERG on this pathway, and thus the necessity of PTEN deletion for increased pathway activation, varies by cellular background. In summary, the cell line profiling presented here provides a basis for using these lines to model the complex crosstalk of oncogenic ETS expression and signaling in various prostate tumors.

The requirement of AKT for transcriptional activation by an ETS factor is novel. This could be due to AKT directly phosphorylating ETS or AP-1 at ETS/AP-1 sequences. AKT is known to modify transcription factors, such as those from the FOXO family [38]. It is also possible that AKT is working through downstream signaling factors. We have ruled out mTORC1, but AKT can modify many other signaling proteins. These AKT-regulated proteins include a number of factors specific to neurons, such as the GABA-A receptor [39], Huntingtin [40], and Ataxin1 [41]. Interestingly, one of the normal functions of the “oncogenic” ETS proteins ETV1 and ETV4 is to cause certain neurons to outgrow and invade the spinal cord during development [42,43]. Furthermore, PI3K/AKT signaling [44], and ETV1 and ETV4 expression [45] can both promote survival of neurons in the absence of neuronal growth factors. Therefore, processes that are oncogenic in prostate epithelia could reflect normal synergy between AKT and these ETS factors in neurons.

The ability to switch the signaling pathway that controls prostate cell migration by altering expression of oncogenic ETS transcription factors provides an interesting example of a mechanism for modulating a gene expression program. Cells can change transcription factor activity via expression levels, or localization. This can gradually alter the fraction of time that a transcription factor occupies a binding site compared to a competing transcription factor. If these competing factors respond to distinct signaling pathways, the effect of this process will depend on the status of each pathway. This allows both transcription factors and signaling pathways to have distinct functions in different cellular backgrounds. In the case of prostate cancer, this work indicates that oncogenic ETS status may be an important factor when deciding to target RAS/ERK or PI3K/AKT signaling during treatment.

## Conclusions

Here we demonstrate that the aberrant expression of an oncogenic ETS transcription factor in prostate cells can switch the regulation of a cell migration gene expression program from RAS/ERK to PI3K/AKT control. This provides a mechanistic rationale for the correlation between PI3K signaling and ERG expression in prostate tumors and identifies a novel mode of ETS regulation that might be exploited by future therapeutics.

## Methods

### Cell culture and viral transduction

All cell lines were authenticated by the University of Arizona Genetics Core using PowerPlex 16HS Assay (Promega) with > 80% match to eight core STR loci [46], with the exception of LNCaP, which was obtained from ATCC immediately prior to use. Cell lines were cultured according to ATCC recommendations as follows; RWPE (RWPE-1) and RWPE-KRAS (RWPE-2): Keratinocyte SFM (Invitrogen), LNCaP and CWR22Rv1: RPMI 1640 (Mediatech-Cellgro) with 10% fetal bovine serum (FBS) [Sigma], PC3: F12K medium (Mediatech-Cellgro) with 10% FBS. 293 EBNA, HEK-293 T, DU145 and VCaP: Dulbecco’s modification Eagle (DMEM) [Sigma] with 10% FBS, MDA-PCa-2b: BRFF-HPC1 (Athena Enzyme Systems) with 20% FBS. All media were supplemented with 1% Penicillin/Streptomycin (Mediatech-Cellgro).

ETS proteins with N-terminal 3xFlag tags were stably expressed in RWPE via retrovirus as described previously [15]. Plasmids for lentiviral shRNA knockdowns were obtained from AddGene, mTOR (#1855), Raptor (#1857) and Rictor (#1853), are from Sarbassov *et al.*[33]. Lentivirus was produced by co-transfection of pLKO.1 constructs in HEK293T cells with pMDLg/pRRE, pRSV-Rev and pMD2.G envelope plasmids from Dull *et al.*[47] and AddGene.

### Transwell migration and *In vitro* scratch assays

Transwell migration assays were done as described previously [15]. In brief, 5×10^4^ cells were introduced to the transwell (8 μM pore size; BD Bioscience) and incubated for 63 h, except for RWPE-KRAS cells summarized in Figure 2C, which were incubated for 54 h. Migrated cells are reported as the mean of four representative fields per membrane, and the mean of two technical replicates (membranes) per biological replicate.

For *in vitro* scratch assays, cells were plated in 35 mm plates and grown to full confluence, and the cultures were scratched by pipette tip. Migration into the open area was documented at 40 h post-scratching by microscopy. Free area was measured using TScratch software (cse-lab.ethz.ch/software) [48].

### Measuring protein and RNA

RNA levels were measured by quantitative reverse transcription-PCR as described previously [15], using primers in Additional file 4: Table S1.

Whole-cell extracts of equivalent cell number were separated by SDS-PAGE and blotted to nitrocellulose. Antibodies for immunoblotting were: ERK (sc-94) and pERK (sc-7383) from Santa Cruz Biotechnology, ETV5 (ab102010) and ETV1 (ab81086) from Abcam, pAKT (S473) and pMEK (9121) from Cell Signaling, Tubulin (T-9026) from Sigma, ETV4 (ARP32263_P050) from Aviva Systems Biology, and ERG (9FY) from Biocare Medical.

Purification of His-tagged ETS proteins for antibody validation was as described previously [49]. DNA binding activity was verified by EMSA. Concentration was calculated by comparison to BSA standards on Coomassie stained 10% SDS-PAGE gels.

### Luciferase assays

Luciferase assays used a Dual Luciferase Reporter Assay System (Promega) according to manufacturer instructions with some modifications. Wild type and mutant ETS/AP-1 sequences (Additional file 4: Table S1) were cloned upstream of the firefly luciferase-pGL4.25 (Promega) plasmid cut with HindIII and NheI. The *Renilla* luciferase gene was sub-cloned from pRL-null to pGL4.25 plasmid by replacing firefly sequence. Cells were plated at ~50% confluency in a 6 well plate (2.5×10^5^ cells/well) 24 hrs before transfection. Cells were transfected with 1 μg of firefly and renilla plasmid using TransIT Prostate Transfection Kit (Mirus). After 24 hours, media was removed, cells were resuspended in 250 μL 1×PLB, and disrupted by three freeze/thaw cycles. Luciferase activity was measured in 20 μL of cell lysate using Appliskan Multimode Microplate reader (Thermo Scientific). Firefly values were normalized to renilla values.

## Abbreviations

RAS/ERK: RAS/RAF/MEK/ERK pathway; pERK: Phosphorylated ERK; pAKT: phosphorylated AKT.

## Competing interests

The authors declare that they have no competing interests.

## Author’s contributions

NS did the experiments reported in Figures 1, 3, and 5. JAB carried out expression profiling and reporter assays in Figure 4. MWF did the migration assays in Figure 2. TJJ interpreted data and assisted in writing. PCH interpreted data and wrote the paper. All authors read and approved the final manuscript.

## Supplementary Material

Additional file 1: Figure S1 Quantitative assessment of ETS protein levels. Click here for file

Additional file 2: Figure S2 Representative images cell migration assays. Click here for file

Additional file 3: Figure S3 Representative images of scratch assays. Click here for file

Additional file 4: Table S1 Oligonucleotide primer sequences. Click here for file
