# Supplementary material for: Prostate cancer ETS rearrangements switch a cell migration gene expression program from RAS/ERK to PI3K/AKT regulation
Source: Mol Cancer. 2014 Mar 19;13:61. doi: 10.1186/1476-4598-13-61 (PMC3999933; doi:10.1186/1476-4598-13-61)
Supplement: Additional file 1: Figure S1 — Quantitative assessment of ETS protein levels. [file 1476-4598-13-61-S1.pdf]

Figure S1

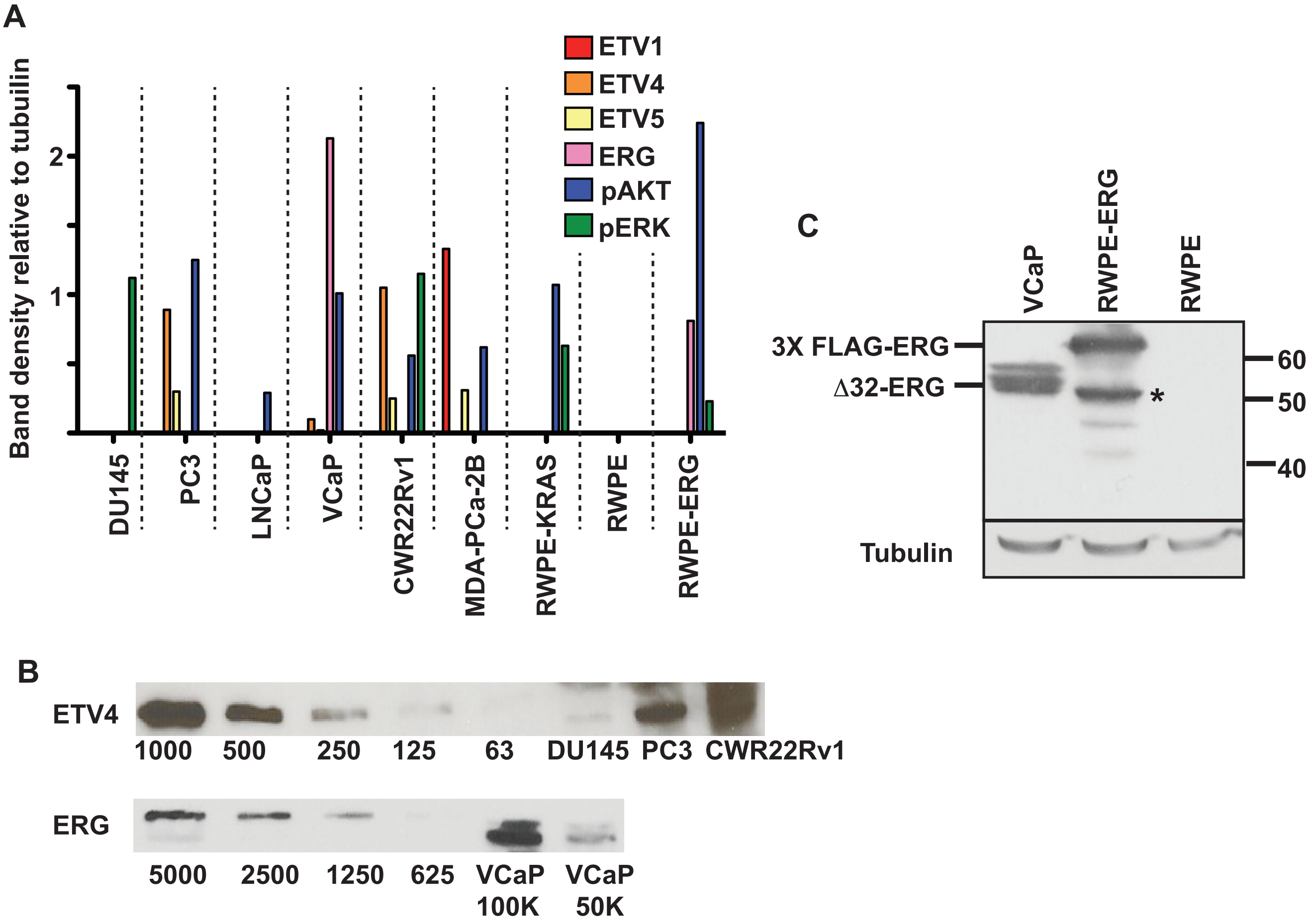

Cell Line	Protein	Estimated Picograms	# Cells Loaded	Molecules Per Cell
CWR22Rv1	ETV4	1000	100,000	112,000
PC3	ETV4	500	100,000	56,000
DU145	ETV4	90	100,000	10,000
VCaP	ERG	1500	50,000	330,000

Figure S1. (A) The density of bands shown in Figure 1B were measured using Image J and are plotted relative to the tubulin levels in the same sample. Note that measurements of bands from the same antibody are comparable within the same blot, but not between blots. Columns 1-6 comprise one blot and 7-9 a second blot. (B) Protein levels of ETV4 and ERG were assayed by immunoblots using the indicated antibody. Comparisons were made between samples containing the indicated number of picograms of purified protein or cell extract from 100,000 cells of the indicated cell type (except 50,000 VCAP cells where indicated). The calculation for approximate number of molecules per cell is shown below. (C) An immunoblot with anti-ERG antibody shows the relative level of truncated (Δ32) ERG expressed from the TMPRSS2/ERG fusion gene in VCaP cells compared to recombinant 3xFLAG full length ERG in RWPE-ERG cells. The asterisk indicates an ERG degradation product. A tubulin loading control for each extract is shown below.
