# Supplementary material for: Prostate cancer ETS rearrangements switch a cell migration gene expression program from RAS/ERK to PI3K/AKT regulation
Source: Mol Cancer. 2014 Mar 19;13:61. doi: 10.1186/1476-4598-13-61 (PMC3999933; doi:10.1186/1476-4598-13-61)
Supplement: Additional file 2: Figure S2 — Representative images cell migration assays. [file 1476-4598-13-61-S2.pdf]

Figure S2

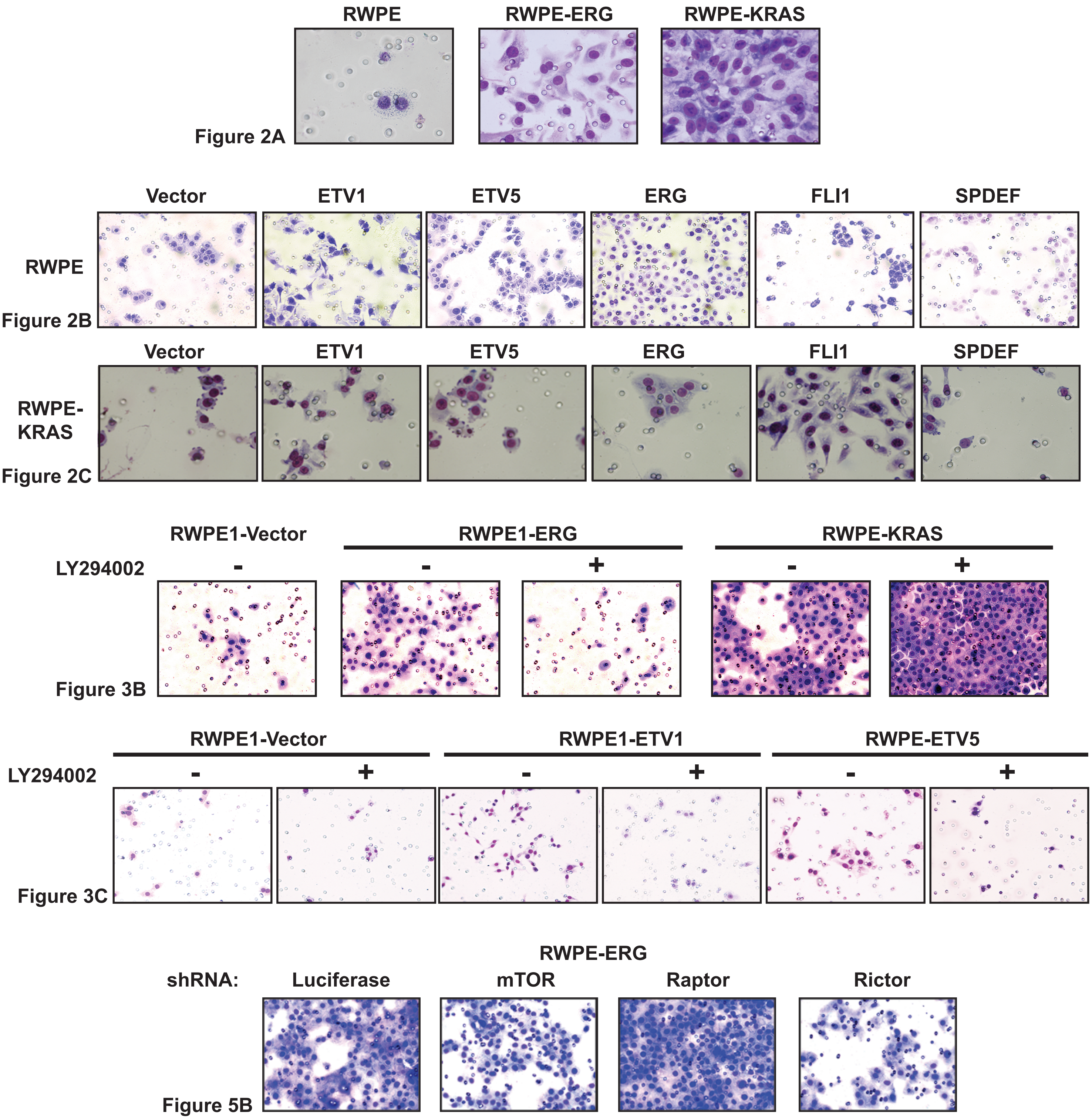

Figure S2. Representative images of transwell migration assays. Cells that migrated through the 8 micron pores were stained and imaged. Cells from four such fields are counted for each technical replicate, and two technical replicates are averaged for each biological replicate. Cells were incubated in transwells for 63 hours, except for RWPE-KRAS cells summarized in Figure 2C, which were incubated for 54 hours, to provide room for increased migration.
