# Supplementary material for: Prostate cancer ETS rearrangements switch a cell migration gene expression program from RAS/ERK to PI3K/AKT regulation
Source: Mol Cancer. 2014 Mar 19;13:61. doi: 10.1186/1476-4598-13-61 (PMC3999933; doi:10.1186/1476-4598-13-61)
Supplement: Additional file 3: Figure S3 — Representative images of scratch assays. [file 1476-4598-13-61-S3.pdf]

Figure S3

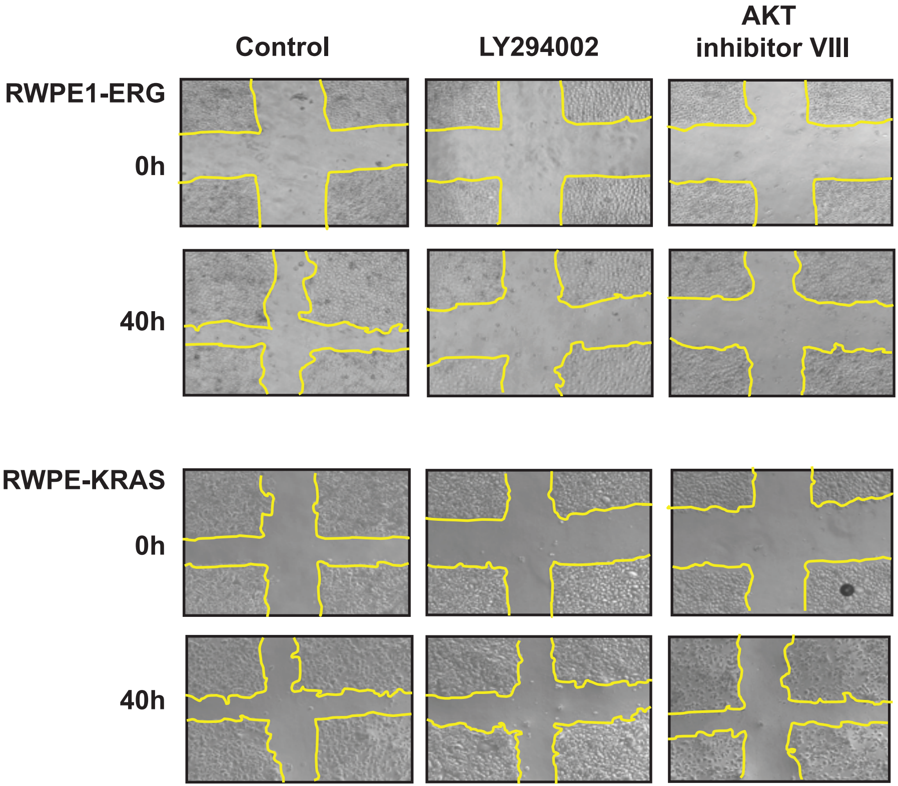

Figure S3. Representative images of a scratch assay performed in RWPE-ERG and RWPE-KRAS cell lines. The cell lines were treated with or without LY294002 (20 μM) or AKT inhibitor VIII (10 μM) and images were taken 0 and 40h post scratching.
