# Supplementary material for: Prostate cancer ETS rearrangements switch a cell migration gene expression program from RAS/ERK to PI3K/AKT regulation
Source: Mol Cancer. 2014 Mar 19;13:61. doi: 10.1186/1476-4598-13-61 (PMC3999933; doi:10.1186/1476-4598-13-61)
Supplement: Additional file 4: Table S1 — Oligonucleotide primer sequences. [file 1476-4598-13-61-S4.pdf]

Table S1

Primer sequences used[a]

RT-PCR SMAD3 forward	GCAGAACGTCAACACCAAGTGC
RT-PCR SMAD3 reverse	GTGCAGGTCTGGCCATCGC
RT PCR SMAD3 reverse transcription	CGTCCTTCTTCATATTGAAGGCG
RT-PCR ARHGAP29 forward	GGAATCAGAACGCAAGCAAAATGCG
RT-PCR ARHGAP29 reverse	GGGATGCTGATTCAGCCTCTTGG
RT-PCR mTOR forward	CTAAGTCTACCACGACAGCCCGG
RT-PCR mTOR reverse	GGCCTTCATGCCACATCTCATGCC
RT-PCR Rictor forward	CAACTGGGATGCTGTGAGGCATAG
RT-PCR Rictor reverse	GTACTAGTAGAGCTGCTGCCAAAC
RT-PCR Raptor forward	GAGAAGCTCTACAGCCTCCTCTCC
RT-PCR Raptor reverse	CCGTCCTCTCTGCAGAGTTGCC
Reporter cloning, 3x ETS/AP-1 forward	CTAGCCACGGCCC**AGGAAGTGACTCA**CTAGC**AGGAAGTGACTCA**AGCTC**AGGAAGTGACTCA**C
Reporter cloning, 3x ETS/AP-1 5' reverse	AGCTGTGAGTCACTTCCTGAGCTTGAGTCACTTCCTGCTAGTGAGTCACTTCCTGGGCCGTGG
Reporter cloning, 3x ETS mt/AP-1 forward	CTAGCCACGGCCC**AGagAGTGACTCA**CTAGC**AGagAGTGACTCA**AGCTC**AGagAGTGACTCA**C
Reporter cloning, 3x ETS mt/AP-1 reverse	AGCTGTGAGTCACTctCTGAGCTTGAGTCACTctCTGCTAGTGAGTCACTctCTGGGCCGTGG
Reporter cloning, 3x ETS/AP-1 mt forward	CTAGCCACGGCCC**AGGAAGTagCctA**CTAGC**AGGAAGTagCctA**AGCTC**AGGAAGTagCctA**C
Reporter cloning, 3x ETS/AP-1 mt reverse	AGCTGTagGctACTTCCTGAGCTTagGctACTTCCTGCTAGTagGctACTTCCTGGGCCGTGG
Reporter cloning, 3x ETS mt/AP-1 mt forward	CTAGCCACGGCCC**AGagAGTagCctA**CTAGC**AGagAGTagCctA**AGCTC**AGagAGTagCctA**C
Reporter cloning, 3x ETS mt/AP-1 mt reverse	AGCTGTagGctACTctCTGAGCTTagGctACTctCTGCTAGTagGctACTctCTGGGCCGTGG

[a] All sequences are listed in 5' to 3' direction, reverse PCR primers were used for reverse transcription, except for SMAD3.
